# Influence of Particulate Matter during Seasonal Smog on Quality of Life and Lung Function in Patients with Chronic Obstructive Pulmonary Disease

**DOI:** 10.3390/ijerph16010106

**Published:** 2019-01-02

**Authors:** Chaicharn Pothirat, Warawut Chaiwong, Chalerm Liwsrisakun, Chaiwat Bumroongkit, Athavudh Deesomchok, Theerakorn Theerakittikul, Atikun Limsukon, Pattraporn Tajaroenmuang, Nittaya Phetsuk

**Affiliations:** Division of Pulmonary, Critical Care and Allergy, Department of Internal Medicine, Faculty of Medicine, Chiang Mai University, 110 Inthavaroros Rd. Sriphum, Maung Chiang Mai, Chiang Mai 50200, Thailand; warawut.chai@cmu.ac.th (W.C.); chalermliw@hotmail.com (C.L.); cbumroon@gmail.com (C.B.); adeesomc@yahoo.co.th (A.D.); theerakorn15@yahoo.com (T.T.); atikun.limsukon@gmail.com (A.L.); pat_taj99@hotmail.com (P.T.); phetsukn@gmail.com (N.P.)

**Keywords:** chronic obstructive pulmonary disease, pollution, lung function, quality of life, dyspnea

## Abstract

The impact of outdoor air pollution on the quality of life (QoL) of chronic obstructive pulmonary disease (COPD) patients, as measured by the COPD assessment test (CAT) questionnaire, is limited. The aim of this study was to determine the impact of a short-term increase in outdoor particulate matter in which the particles are less than 10 microns in diameter (PM_10_) during a seasonal smog period on QoL, symptoms, and lung function in COPD patients. This prospective observational study was conducted at Chiang Dao Hospital, Chiang Mai, Thailand between March and August 2016. Measurement of QoL, severity of dyspnea, forced vital capacity (FVC), and forced expiratory volume in the first second (FEV_1_) were performed at both high and low PM_10_ periods. Fifty-nine patients met the inclusion criteria for enrollment into the study, with the mean age being 71.5 ± 8.0 years. Total CAT score, but not mMRC score, was statistically higher during the high PM_10_ period. The two lung function parameters, FVC and FEV_1_, were significantly lower at the high PM_10_ compared to the low PM_10_ period. We concluded that exposure to PM_10_ during the seasonal smog period resulted in short-term negative impact on the quality of life and lung function in COPD patients.

## 1. Introduction

Chronic obstructive pulmonary disease (COPD) is predicted to become the fourth leading cause of death worldwide by 2030 [1]. The most significant cause of COPD, after chronic cigarette smoking, is exposure to biomass smoke, especially in developing countries [2]. In Asia-Pacific countries and regions, the estimated prevalence of COPD ranges from 3.5% to 6.7% [3], and the prevalence of COPD in Chiang Dao was 6.8%, which was twice as high as that of Chiang Mai shown in a recent study [4]. 

Chiang Dao, with latitude and longitude of 19°21′58′′N 98°57′51′′E, is a district in Chiang Mai Province in Northern Thailand surrounded by high mountain ranges and covering an area of approximately 1882 km^2^. It has a population of around 87,992 people distributed among 7 sub-districts. Due to its geographical features, Chiang Dao has been exposed to air pollution, especially during the dry season, for many years. Agricultural burning and forest fires in Chiang Mai Province has also contributed to the seasonal smog crisis between January and April each year since 2006 [5].

Particulate matter with particles less than 10 microns in diameter (PM_10_) is a pollutant that is known to adversely affect human health. PM_10_ presents a wide variety of constituents, such as metals and trace elements, organic compounds, and acids [6]. PM_10_ has been reported to be a significant factor in the exacerbation of respiratory diseases including asthma and COPD [5,7,8]. Previous studies have found that short-term exposure to outdoor air pollution, especially PM_10_, is associated with increased respiratory symptoms [7], decreased lung function [8,9,10,11], and acute exacerbation [5] in COPD patients. However, these studies did not determine the impact of PM_10_ on QoL. 

It has been demonstrated that there is a strong relationship between quality of life (QoL) and the number of acute exacerbations of COPD [12]. To our knowledge, there are limited studies investigating the effects of air pollution on QoL in patients with COPD; however, their results are inconsistent [13,14]. The COPD assessment test (CAT) is one of the health-related quality of life (HR-QoL) instruments recently verified for COPD [15], and the northern Thai version of CAT has already been recommended for the local setting [16]. However, knowledge regarding the effect of atmospheric air pollution on the QoL of COPD patients determined by the CAT questionnaire is still limited. This study therefore aimed to determine the effect of increased outdoor PM_10_ on QoL and lung function in COPD patients living in the Chiang Dao district of Chiang Mai, Thailand.

## 2. Materials and Methods

### 2.1. Study Design and Population

A prospective observational study was conducted between March and August 2016 in Chiang Dao district. Ninety COPD patients were screened for eligibility at the outpatient department of Chiang Dao Hospital, Chiang Mai, Thailand. The recruitment criteria included: patients aged over 40 years with a diagnosis of COPD based on post-bronchodilator (BD) ratio of forced expiratory volume in the first second (FEV_1_)/forced vital capacity (FVC) <0.7 according to the Global Initiative for Chronic Obstructive Lung Disease (GOLD) criteria [17]; ex-smokers with a smoking history of more than 10 pack-years; no history of acute exacerbation (AE) for at least three months prior to the enrollment; and those receiving long term pharmacological treatment for COPD. Patients meeting any of the following criteria were excluded: current diagnosis of asthma; current active respiratory disorders other than COPD, e.g., lung cancer, tuberculosis, or other significant chest X-ray findings not associated with COPD (documented within the past 1 year). Fifty-nine COPD patients were included in this study. The study was approved by the Ethics Committee of the Faculty of Medicine, Chiang Mai University (Study code: MED-2558-03032, Date of approval: 14 December 2015)

### 2.2. Measurements of Air Pollutants (PM_10_) and Meteorological Parameters

Ambient air concentration of pollutants was measured by Dust DETECT^TM^ at the sampling station located in Chiang Dao hospital, Chiang Mai, Thailand. The analytical method for PM_10_ was specifically designed to monitor the flow of particulate emissions from small stacks and emission points while passing through an air filtration system. The maximum, minimum and daily average of PM_10_ data and meteorological data including temperature, relative humidity, rainfall, wind speed, and pressure were collected for on the months of March and August 2016. The PM_10_ during March was denoted the high PM_10_ period and that during August was denoted the low PM_10_ period. All meteorological data from the monitoring stations in Chiang Dao district is available from https://meteorology.hrdi.or.th. 

### 2.3. Outcome Measures

Measurements were collected twice: in March 2016 and in August 2016. Data collection included QoL, dyspnea severity, and post-bronchodilator pulmonary function test. 

#### 2.3.1. Pulmonary Function Test

All subjects were evaluated for FVC, FEV_1_, ratio of FEV_1_/FVC and, forced expiratory flow at 25–75% (FEF_25–75%_) using a spirometer (Spiromaster PC-10, CHEST M.I., Inc. Tokyo, Japan) following the standard guidelines recently published by the American Thoracic Society (ATS)/European Respiratory Society (ERS) [18]. Predicted values were calculated using the Knudson reference equation [19].

#### 2.3.2. Quality of Life and Dyspnea Severity

The CAT questionnaire, designed to assess the health status of COPD patients, has 8 items covering cough, phlegm, chest tightness, breathlessness when walking up a hill or one flight of stairs, limitation in doing activity, reduced self-confidence, sleep disturbance, and loss of energy. Each item is scored from 0 to 5 to give a total score ranging from 0 to 40, corresponding to the best and worst health status in patients with COPD, respectively [15]. The northern Thai version of CAT [16] was administered to all participating subjects. Dyspnea severity was classified using the modified Medical Research Council (mMRC) dyspnea scale [20].

### 2.4. Statistical Analysis

Results for numerical values were expressed as mean ± standard deviation (SD) or median (Interquartile range, IQR) and number (%) for categorical variables. Different values of pollutant data, CAT score, and lung function between low and high PM_10_ periods were determined using paired *t*-tests. A different value of mMRC scores was determined using a Wilcoxon signed rank test. Statistical significance was set at a *p*-value < 0.05. All analyses were carried out using the SPSS statistical package, version 22 for IBM (IBM Corp., Armonk, NY, USA).

## 3. Results

### 3.1. Patient Characteristics

Out of 90 patients assessed for eligibility, 31 were excluded due to current diagnosis of asthma (*n* = 14), diagnosis unmet for COPD following GOLD criteria (*n* = 15), and inability to perform spirometry (*n* = 2). The fifty-nine patients meeting the inclusion criteria for enrollment (31 men, 52.5%) had a mean age of 71.5 ± 8.0 years, mean body mass index (BMI) of 20.2 ± 4.1 kg/m^2^, and mean %predicted of FEV_1_ of 64.2 ± 24.3. According to GOLD classification [17], 13 (22.0%) cases were in GOLD A, 17 (28.8%) in GOLD B, 14 (23.7%) in GOLD C, and 15 (25.4%) in GOLD D categories. All the participants were ex-smokers living in open housing style homes, indicating that they were exposed to atmospheric air 24 h a day. The baseline characteristics of patients in this study are shown in Table 1. 

### 3.2. Pollutant Data 

Maximum, minimum, and average PM_10_ were significantly higher in March 2016 (High PM_10_ period) compared to August 2016 (Low PM_10_ period). Atmospheric pressure was also significantly higher during the high PM_10_ period when compared to the low PM_10_ one. In contrast, humidity was significantly lower during the high PM_10_ period compared to the low PM_10_ period. Temperature, rainfall, and wind speed were not statistically different between the high and low PM_10_ periods. The pollutant data are summarized in Table 2.

### 3.3. Quality of Life and Dyspnea Severity during the High PM_10_ versus the Low PM_10_ Period

Total CAT scores were significantly higher in March 2016 (High PM_10_ period) compared to August 2016 (Low PM_10_ period) (11.7±7.1 vs. 9.6 ± 5.6, *p* = 0.013). In the subdomains of the CAT questionnaire, chest tightness and sleep disturbance were also statistically higher in the high PM_10_ period when compared to the low PM_10_ period (1.2 ± 1.3 vs. 0.7 ± 1.1, *p* = 0.016 and 0.8 ± 1.2 vs. 0.4 ± 0.8, *p* = 0.016 respectively). However, the other subdomains of CAT questionnaire were not statistically different between the high and low PM_10_ periods including the dyspnea severity. The difference in QoL and dyspnea severity between the months of high PM_10_ and low PM_10_ are shown in Table 3.

### 3.4. Lung Function in the Period of High PM_10_ versus the Period of Low PM_10_

FVC and FEV_1_ were significantly lower in March 2016 (High PM_10_ period) when compared to August 2016 (Low PM_10_ period) (2.07 ± 0.68 vs. 2.14 ± 0.68, *p* = 0.025 and 1.19 ± 0.48 vs. 1.25 ± 0.51, *p* = 0.008 respectively). However, there was no statistically significant difference in FEF_25–75%_ data between March 2016 (High PM_10_ period) and August 2016 (Low PM_10_ period). The differences in lung function between the months of high PM_10_ versus low PM_10_ are shown in Table 4.

## 4. Discussion

The observational study conducted in Chiang Mai, Thailand, revealed poor QoL, particularly in the form of chest tightness and sleep disturbance as well as a decreased lung function during the high PM_10_ period in patients with COPD. The latter was supported by previously published studies where decrements in both FVC and FEV_1_ were associated with increasing pollution concentration in patients with COPD [9,10]. Our results on the decrease in FEV_1_ of about 60 milliliters between the high and low PM_10_ period was twice that of an earlier study which reported a 30 milliliters/year decline in FEV_1_ [21]. In our study, the FEF_25–75%_ did not show a statistically significant decrease during the high PM_10_ period, which probably reflects the impact of a chronic disease, as subjects with COPD may have difficulty with this effort-dependent maneuver. Therefore, in these subjects, FEV_1_ appears to be a more robust parameter than small airway function tests. Additionally, FEF_25–75%_ is dependent on the FVC, and therefore, changes in FVC will affect the portion of the flow-volume curve examined. If FEF_25–75%_ is not adjusted for lung volume, there is poor reproducibility [22]. 

There was a significant decrease in QoL when measured by total CAT score during the high PM_10_ period, especially in the form of chest tightness and sleep disturbance subdomains. However, there were no significant increases in cough, phlegm, and physical activity limitation associated with PM_10_ in our study, in contrast to previous reports [7,23,24]. 

The exact mechanism by which PM_10_ may influence lung function is uncertain [25]. However, previous studies suggest that PM_10_ may mediate adverse health effects via the generation of reactive oxygen species (ROS), activation of cell signaling pathways, and alterations of respiratory tract barrier function and antioxidant defenses, all of which may lead to airway inflammation and changes in pulmonary function [26,27,28]. In cases of COPD, these factors could explain the activation of inflammatory mechanisms causing tissue damage and subsequently increasing the sensitivity of the trachea leading to the decrements in lung function. In addition, a previous review on the role of PM_10_ as a cause of oxidative stress enhancing pro-inflammatory effects in the airway of patients already activated by the disease [29], might explain the aggravation of symptoms in patients with COPD, specifically chest tightness and sleep disturbances.

The major strength of our study was that we selected a very specific population of COPD, therefore limiting extraneous variables as much as possible. The population included Chiang Dao dwellers living in areas exposed to seasonal smog during the entire study period. All of them were ex-smokers and were exposed to atmospheric air pollution at all times. However, our study has several limitations. Firstly, a time series analysis to assess the trends and relationships using generalized estimation equation with Poisson regression analysis could not be executed due to the nature of the data collected. Secondly, the other pollutants, including SO_2_, NO_2_, CO, and O_3_, as well as the temperature and humidity, could not be adjusted. Thirdly, the influence of other pollutants, including SO_2_, NO_2_, CO, and O_3_, or other metrological data, on QoL and lung function could not be demonstrated due to limited data.

## 5. Conclusions

This study suggests that there is a short-term negative impact of exposure to PM_10_ on QoL and lung function in patients with COPD. Worsening of QoL, particularly chest tightness and sleep disturbance and decrements in lung function (FVC and FEV_1_), were shown during the high PM_10_ period. These results might provide useful information for improving the health of COPD patients during periods of high air pollution.

## Figures and Tables

**Table 1:** Baseline characteristics of patients in this study.

Variables	All Patients (*n* = 59)
Age (years)	71.5 ± 8.0
Male sex, *n* (%)	31 (52.5)
BMI (kg/m^2^)	20.2 ± 4.1
Smoking pack-year	36.0 ± 38.8
Pulmonary function data	
FVC	2.07 ± 0.68
Percent predicted of FVC	89.0 ± 25.9
FEV_1_	1.19 ± 0.48
Percent predicted of FEV_1_	64.2 ± 24.3
Ratio of FEV_1_/FVC (%)	57.0 ± 10.2
GOLD classification, N (%)	
A	13 (22.0)
B	17 (28.8)
C	14 (23.7)
D	15 (25.4)
Hx. of AECOPD	16 (27.1)
No. of AECOPD (pt/year)	2.4 ± 2.5
Charlson comorbidity index	3.86 ± 1.04
Medication used	
SABA	42 (71.2)
Oral beta-2 agonist	32 (54.2)
LTOT	1 (1.7)
Ex-smoker	59 (100.0)
Open-housing style	59 (100.0)

**Notes:** Results are expressed as mean ± SD; GOLD A was defined as low symptom severity and low exacerbation risk; GOLD B was defined as high symptom severity and low exacerbation risk; GOLD C was defined as low symptom severity and high exacerbation risk; GOLD D was defined as high symptom severity high exacerbation risk; low symptom severity is considered a CAT score less than or equal to 9, high symptom severity is considered a CAT ≥ 10; low risk of exacerbation is defined as no more than one exacerbation not resulting in hospital admission in the last 12 months; high risk of exacerbation is defined as at least two exacerbations or any exacerbations resulting in hospital admission in the last 12 months. **Abbreviations:** BMI, Body mass index; FVC, forced vital capacity, FEV_1_, forced expiratory volume in first second; AECOPD, acute exacerbation of COPD; SABA, short acting beta-2 agonist; LTOT, long term oxygen therapy.

**Table 2:** Pollutant data between the high PM_10_ period (March 2016) and the low PM_10_ period (August 2016).

Variables	Low PM_10_ (August 2016)	High PM_10_ (March 2016)	*p*-Value
PM_10_ (µg/m^3^) Max (range)	40.9 (31.3–45.4)	215.8 (149.1–268.5)	0.001
PM_10_ (µg/m^3^) Min (range)	13.3 (10.5–24.3)	51.7 (28.1–80.8)	0.034
PM_10_ (µg/m^3^) Mean(range)	29.2 (18.4–32.4)	120.4 (82.3–149.2)	0.003
Temperature (°C)	25.8 (25.4–26.1)	25.8 (24.1–26.4)	0.248
Rainfall (mm)	0.0 (0.0–0.0)	0.0 (0.0–0.0)	0.076
Wind speed (km/h)	33.4 (20.4–51.9)	37.1 (31.5–40.8)	0.800
Humidity (%)	67.5 (66.8–68.3)	61.8 (61.2–62.2)	<0.001
Pressure (millibar)	1004.5 (1002.9–1006.7)	1014.8 (1021.1–1015.3)	0.028

**Note:** Data are median (IQR); **Abbreviations:** IQR, interquartile range; PM_10_, Particulate matters with diameter of less than 10 micron; m^3^, per cubic meter.

**Table 3:** Quality of life and dyspnea in the period of high PM_10_ versus the period of low PM_10_.

Variables	Low PM_10_	High PM_10_	*p*-Value
CAT total score	9.6 ± 5.6	11.7 ± 7.1	0.013 *
Cough	1.6 ± 1.0	1.7 ± 1.0	0.756
Phlegm	1.4 ± 1.2	1.5 ± 1.2	0.492
Chest tightness	0.7 ± 1.1	1.2 ± 1.3	0.016 *
Breathless when walk up a hill or one flight	1.6 ± 1.2	1.8 ± 1.4	0.395
Limit doing activity	1.1 ± 1.2	1.5 ± 1.3	0.098
Self confidence	1.0 ± 1.3	1.3 ± 1.7	0.379
Sleep disturbance	0.4 ± 0.8	0.8 ± 1.2	0.016 *
Loss of energy	1.8 ± 1.2	2.0 ± 1.2	0.370
mMRC (median, IQR)	1 (1–2)	1 (1–3)	0.465

**Notes:** Results are expressed as mean ± SD or median (IQR); *, statistical significance. **Abbreviations:** CAT, COPD assessment test; mMRC, modified medical research council score

**Table 4:** Lung function in the period of high PM_10_ versus the period of low PM_10_.

Lung Function Data	Low PM_10_	High PM_10_	*p*-Value
FVC (L)	2.14 ± 0.68	2.07 ± 0.68	0.025 *
FEV_1_ (L)	1.25 ± 0.51	1.19 ± 0.48	0.008 *
FEF_25–75%_ (L)	0.64 ± 0.38	0.59 ± 0.31	0.122

**Notes:** Results are expressed as mean ± SD; *, statistically significant. **Abbreviations:** FVC, forced vital capacity; FEV_1_, forced expiratory volume in first second; FEF_25–75%_, forced expiratory flow at 25–75% of FVC.
